# Hospital Meetings, &c.

**Published:** 1897-03-27

**Authors:** 


### The Institutional Workshop.

### HOSPITAL MEETINGS, &C.

### ASSOCIATION FOR THE ORAL INSTRUCTION OF THE DEAF AND DUMB.

The Duke of York presided over the festival dinner in aid of the Association for the Oral Instruction of the Deaf and Dumb, which was held at the Whitehall Rooms on Wednesday, the 17th inst. Amongst those present were the Duke of Fife (the president of the association), the Swedish, Netherlands, and Belgian Ministers, the Earl of Limerick, Viscount Doneraile, the Hon. D. Keppel, Sir Francis de Winton, Sir Frederick Milner, Bart., M.P., Admiral Markham, and Sir Henry Thompson. The Duke of York, in proposing the toast of the evening, "Prosperity to the Association for the Oral Instruction of the Deaf and Dumb," said that the institution was founded about thirty years ago by the late Baroness Meyer de Rothschild. The form of instruction pursued was a great improvement on the old system of the language of signs, for those who had been instructed in the principles of the new method were able to hold conversation with strangers, while before they could only converse with those who knew the finger alphabet. A few days before he had visited the schools of the association, and he was astonished at the rapidity with which the children answered the questions of the teachers, and the great intelligence that nearly all of them showed. They were, he said, almost as quick with answers to the questions put by those present as they were to the teachers. To pay off the present liabilities, and to carry out certain improvements, a sum of £7,000 was required. The Duke of Fife responded on behalf of the association. Subscriptions amounting to £3,300 were announced as the result of the dinner.

### WEST END HOSPITAL.

The annual general meeting of the governors of the West End Hospital for Diseases of the Nervous System, &c., Welbeck Street, was held at the hospital on Thursday, the 18th inst., Major-General T. W. Mercer (chairman of the committee of management) in the chair. On the motion of the Chairman, seconded by Mr. F. Rowney, the report was adopted. The ordinary receipts in 1896 amounted to £2,575 and the ordinary expenditure to £2,841. The hospital had also received a donation, for the purpose of naming a ward and cot, of £1,000. The balance in hand, exclusive of this donation, amounted to £269. 272 new in- and 3,956 new out-patients had been treated during the year, their payments amounting to £95 and £401 respectively. The retiring members of the committee of management and other officers were re-elected, and votes of thanks to the honorary medical staff, the treasurer, and the chairman were passed.

### METROPOLITAN HOSPITAL.

The Lord Mayor took the chair at the annual festival dinner of the Metropolitan Hospital, which was held on Thursday, the 18th inst., at the Whitehall Rooms. Although situated in the midst of a very poor neighbourhood, with no other general hospital—except the German Hospital—within two miles of it, not quite one-half of the 160 beds which the hospital can provide, have ever been opened and furnished, as the funds have not been forthcoming for this purpose. The 70 beds which are open at present have to meet the requirements of a district which numbers close upon half a million. Mr. Leopold de Rothschild, who has recently accepted the office of treasurer, in responding to the toast of "Prosperity to the Hospital," called attention to the good work done to the poor Jews by the hospital. During the past year there had been 170 patients in the Jewish wards, and 4,312 Jews had been treated as out-patients, most of whom hardly knew a word of English, and to whom a special department was devoted. The Secretary announced that the dinner had resulted in a subscription list of just over £2,000.

### UNIVERSITY COLLEGE HOSPITAL.

The annual meeting of the governors of University College Hospital was held on Friday, the 19th inst., in the board-room, Lord Monkswell presiding. Fourteen members of the Hospital Committee were elected by ballot. The report, the adoption of which was moved by the Chairman, and carried, after referring to the special efforts which had been made to strengthen the financial position of the hospital, stated that the daily average number of in-patients treated during 1896 was 188. The number of in-patients was 3,020, and of out-patients was 43,681, these latter including 30,177 casualties. In proposing a vote of thanks to Sir Blundell Maple for his handsome gift of £100,000 to the hospital, the Chairman said that had it not been for that gift it would have been some time before the committee could have seen their way to carrying out the much-needed rebuilding of the hospital. They would have been obliged to rebuild it piecemeal, and they might have hoped to finish one wing within a reasonable period of time, but beyond that their aspirations hardly ventured to soar. Mr. Henry Lucas, chairman of the Hospital Committee, seconded, and explained that the amount which had been collected for a building fund previous to Sir Blundell Maple's gift, had to a large extent, as a condition of that gift, been used to acquire the outstanding leases of the portions of land required to complete the re-erection of the hospital. Votes of thanks to the officers and to the chairman concluded the business.

### LONDON HOSPITAL SATURDAY FUND.

The Lord Mayor presided at the annual public meeting of the London Hospital Saturday Fund, which was held at the Mansion House on Saturday, the 20th inst. The meeting was very well attended, and conspicuous among those present were the members of the Hospital Saturday Fund division of the St. John Ambulance Brigade in uniform, and the nurses who form the nursing section of that division.

The Lord Mayor having briefly opened the proceedings, Mr. R. B. D. Acland, chairman of the Fund, detailed the chief events of the past year. The total receipts for 1896, he said, were the largest that had ever been known in the history of the Fund, and amounted to £21,614. Of that sum £4,884 was collected in the streets, while £16,337 was collected in the workshops and warehouses of London. (Applause.) The latter amount showed an increase over that collected in the year 1894 of something like £1,500. The amount distributed among the medical charities, including the Distribution Committee for the Purchase of Letters, and the Surgical Appliance Committee, was £18,110, and a balance of £974 was carried forward. An ambulance brigade, consisting of two surgeons, two sergeants, and thirteen privates, and a nursing division had been formed during the year.

Mr. Sydney Buxton, M.P., moved the following resolution:—

"That this meeting wishes every success to the Fund inaugurated by the Prince of Wales, and, bearing in mind his Royal Highness's determination not to trench upon the ground occupied by the Hospital Saturday Fund, trusts that all engaged in the workshops of London will, through that organization or otherwise, make a special effort this year to prove that the workers of the metropolis are anxious to take their share in the attempt to put the finances of the hospitals into a satisfactory condition."

Mr. H. N. Hamilton-Hoare seconded, and the resolution was carried.

The Bishop of London proposed a resolution recommending for more general acceptance the principle of collecting small weekly contributions in the workshops of London for the Metropolitan medical charities.

Lord Monkswell seconded, and Dr. T. R. Taylor supported the resolution, which was agreed to.

The Lady Mayoress then distributed the certificates gained by the St. John Ambulance classes held in connection with the Fund, after which a vote of thanks to the Lord and Lady Mayoress concluded the proceedings.

### ROYAL SEA-BATHING INFIRMARY, MARGATE.

The annual Court of Governors of the Royal Sea-bathing Infirmary, Margate, was held at the offices of the charity, 30, Charing Cross, S.W., on Monday, the 22nd inst., Mr. Michael Biddulph, M.P. (Treasurer), in the chair. In moving the adoption of the report, the Chairman compared the work done with the expenditure during the past four years, and submitted the following figures:—

| | 1893. | 1894. | 1895. | 1896. |
|---|---|---|---|---|
| Beds open ... | 80 ... | 84 ... | 94 ... | 123 |
| Patients under treatment ... | 445 ... | 490 ... | 512 ... | 574 |
| Cost of provisions ... | £2,554 ... | £2,533 ... | £2,522 ... | £2,357 |
| Cost of Surgery and Dispensary (including wine) ... ... | £601 ... | £623 ... | £482 ... | £363 |

From these figures he said it would be seen that the value of the institution had not only been increased, but that the expenses had been enormously diminished, owing in a great measure to the reorganisation of the hospital and the placing of everything on a proper and better footing. During the year certain defects in the older portion of the fabric of the infirmary had been dealt with; amongst others, a new operation theatre had been commenced, part of the fittings of the kitchen and laundry had been renewed, and the outside of the entire building had been repainted. The new Archbishop of Canterbury had consented to become a vice-patron.—The report was adopted.—The honorary officers were re-elected, and certain alterations in the laws were agreed to.

### CITY OF LONDON HOSPITAL FOR DISEASES OF THE CHEST.

The festival dinner in aid of the funds of the City of London Hospital for Diseases of the Chest was held on Tuesday, 23rd inst., at the Whitehall Rooms, the Hon W. F. D. Smith, M.P., in the chair. The financial condition of this institution has been a source of anxiety for some time past, and while unwilling to entertain the possibility of being compelled to close wards through want of funds, the committee, in the special appeal they issued on the occasion of the dinner, feared that unless a more than usual success attended the present effort, such a contingency might arise. The amount of the collection as a result of the dinner was over £1,860, a sum about equal to the average received at these dinners during the past four years. Amongst the amounts contributed by the working-men's societies were 260 guineas each from the Bethnal Green Working Men's Society and the Albert and Victoria Society. The Chairman, in proposing "Prosperity to the Hospital," said that one particular feature of the hospital was the very great interest taken in it by the working men of the district it served. Two facts proved this; one was that in the dispensary boxes during the past year no less than £50 was found in pence, and also that during the year £800 was subscribed by working men organisations. The necessity for such a hospital was shown by the number of patients who used the hospital. During the year 1896 938 in-patients and 17,451 out-patients had been admitted.

### ROYAL ALFRED AGED MERCHANT SEAMEN'S INSTITUTION.

The annual meeting of the Royal Alfred Aged Merchant Seamen's Institution was held on Monday, the 22nd inst., in the Shipping Exchange, Billiter Street, Mr. R. S. Donkin, M.P. (president), in the chair. The report stated that during the past year eight aged and destitute seamen had been admitted into the society's home at Belvedere, Kent, and 23 to the receipt of out-pensions. The present number of inmates in the home is 100, while the out-pensioners, each receiving £12 a year, number 236. For the period under review the receipts were £7,360, as against £5,650 for 1895, while the disbursements were £7,142, as compared with £6,555 in the previous year. The report concluded with an earnest appeal for increased support, it being stated that there are 280 approved applicants for the society's benefits, 180 of whom are between 70 and 85 years of age and 60 of whom have stood from 10 to 22 elections. In moving the adoption of the report, the Chairman remarked that, considering the maritime position of this country, he thought it was anything but creditable that so many old and needy sailors should have to be denied the benefits of the institution through lack of funds. Admiral M'Clintock seconded the resolution, and the report was adopted. A vote of thanks to the chairman terminated the proceedings.

### POPLAR HOSPITAL FOR ACCIDENTS.

The annual meeting of governors and subscribers to the Poplar Hospital was held at the hospital on Tuesday, March 23rd, the Hon. Sydney Holland presiding. A particularly friendly and pleasant spirit always pervades these meetings at Poplar, the same keen interest being evidently taken in the hospital and its work alike by the working men governors, who give it such good support, the members of the committee, and of the medical and nursing staffs.

Mr. Holland went through the various items in the report, which stated that annual subscriptions and donations had increased, and legacies had provided money in hand to pay for the new buildings, which are to be opened in May, and contain a larger receiving-room, a good operating theatre, and extra accommodation for the nurses. Two more improvements were yet to come—rooms for the sisters off the wards, and a small chapel, towards which Mr. Campbell, the hon. chaplain, had generously promised £100, but it was hoped some governor would come forward and help also. There was no intention at present of increasing the number of beds, but the hospital was not large enough to treat all the accidents in the neighbourhood, and the establishment of dental and eye departments would add greatly to its good work. They would cost little to keep up, as only ten beds would be needed, but the initial cost would probably be about £10,000, hardly a possible outlay unless some generous person would undertake it for the good of the neighbourhood. During the year accidents had been treated at the rate of over four for every hour of every day. Commenting upon the charge of 2d. now made to out-patients towards the cost of bandages, Mr. Holland said that a falling off in attendances had been prophesied. These had, however, increased from 16,624 in 1895 to 17,479. It was creditable to the working men, who had at first opposed this change, that they had now fallen into line, and the pence had been paid not only willingly and without the slightest grumbling, but many patients had insisted on paying when there was no need for them to do so under the rules. The income to the hospital from this source is said to be permanently increased by about £100 a year. Cordial thanks were tendered to Dr. Freshfield for generous help, to Messrs. Bullivant for a gift of iron fencing for the tennis court, to the hon. medical staff, the nursing staff, and to Mr. Radford (house-surgeon), and the two assistant house-surgeons for their good work during the year. The nursing got better every year, and great thanks were due to Miss Bland for the conscientious and thorough way in which she carried out her work. Mr. Radford was leaving the hospital after four and a half years' devoted service, regretted by everybody. Mr. Holland mentioned his election to the chairmanship of the London Hospital during the year, and said that he had been asked if that would mean his leaving Poplar. He should be very sorry ever to leave Poplar so long as they liked to have him for their chairman, a statement received with much applause.

The adoption of the report was duly proposed, seconded, and carried, and the meeting concluded with the re-election of the committee, and the usual votes of thanks.

